# The musculoskeletal abnormalities of the Similaun Iceman (“ÖTZI”): clues to chronic pain and possible treatments

**DOI:** 10.1007/s10787-012-0153-5

**Published:** 2012-10-25

**Authors:** Walter F. Kean, Shannon Tocchio, Mary Kean, K. D. Rainsford

**Affiliations:** 1 Division of Rheumatology, McMaster University, 401-1 Young Street, Hamilton, ON L8N1T8 Canada; 2 Department of Radiology, University of Pittsburg, Pittsburg, USA; 3 Maclain Consultants Inc., Ontario, Canada; 4 Biomedical Research Centre, Sheffield Hallam University, Sheffield, UK

**Keywords:** Similaun Iceman, Arthritis, Tattoos, Lyme disease

## Abstract

**Background and Introduction:**

In 1991, a deceased human male was found frozen in a glacier pool in the Italian Alps in north west Italy, and is now carefully preserved in the South Tyrol Museum of Archaeology, in Bolzano, Italy. The bodily tissues of the 5,300 year old male (colloquially referred to as the Iceman or Ötzi) were well preserved despite damage related to freezing, and glacial movement. Associated articles of well-preserved clothing, tools, weapons and other devices were also present and have been studied in detail. Clinical examination and imaging investigations have also shown that the Icemen had experienced possible illnesses in his lifetime and had identifiable areas of arthritis and musculoskeletal injury. This report includes some key observations on the musculoskeletal state of Ötzi and reference to the involvement of tattoo markings. Some aspects about the aetiology of his abnormalities and inflammatory arthritis are considered along with possible treatments that he might have employed.

**Methods and results:**

We (WFK and MK) undertook a clinical musculoskeletal examination of the Iceman, details of which with available photographs and radiographic imaging pertaining to the musculoskeletal findings of the Iceman are reported here. The skin of the Iceman has numerous linear carbon tattoos, which are not of a decorative type. These have been presumed to possibly be “medicinal” tattoos administered for therapeutic reasons and may have been used in acupuncture-like treatment of pain. Spinal imaging identified areas of spinal damage and our observations have provided clues as to possible sites of spinal initiated pain and hence sites for administration of the “medicinal” tattoos. We observed body areas of the Iceman, in which imaging demonstrated arthritis and other forms of long-term musculoskeletal damage, but which do not have adjacent or corresponding “medicinal” tattoos. We contend that the back and leg “medicinal” tattoos correspond directly to sites of chronic right knee and right ankle pain, and left thoracolumbar pain. They also correspond to lower lumbar and sciatic referred radicular pain which may have a contributory cause related to the presence of a transitional lumbar 5 vertebra. Using recent published data (Keller et al. in Nature Commun 3:698, 2012. doi:10.1038/ncomms1701) of the genome structure of the Iceman, we suggest some potential causes of the osteoarthritis or inflammatory joint injury may relate to presence of coronary heart disease (CHD) and Lyme disease (*Borrelia burgdorferi)* infection. We speculate on possible medical applications of natural products for self-medication.

**Conclusions:**

These observations highlight several diagnostic features of musculoskeletal conditions in the Iceman with the possibility that tattoos may have been used for diagnosis or location of his painful states. The origins of his musculoskeletal conditions are unclear but there are indications that Lyme disease and CHD may have been factors. The associations or use of natural products may give insights into their applications at the time of the life of the Iceman.

## Introduction

In September 1991 two hikers, Erika and Helmut Simon found a frozen human male deceased specimen in a glacier pool in the Italian Alps north west of Bolzano, Italy. Anthropological studies have estimated that this man was alive at least 5,300 years ago. The bodily tissues of this ancient were very well preserved despite damage related to freezing, and glacial movement. His clothes and possessions including tools, a bow, arrows, other weapons and devices were also very well preserved. Since he was found in the region of the Ötztal Alps and near the Similaun mountain refuge, the male specimen became colloquially and respectfully referred to as the Similaun Iceman or Ötzi. The clothing, tools, weapons and other artifacts have been studied in detail. In addition, scientific experts of numerous disciplines have studied the body tissues, the intestinal contents, and bodily contaminants, and where relevant have subjected these to investigation by gross anatomy, pathology, histology, bacteriology, biochemistry, and DNA structure. There exists an excellent knowledge base on the Iceman’s region of origin, habitat, activities, work habits, and the food contents in his last few days and hours of life (Fleckinger 2005; Oeggl et al. 2007). He was estimated to be about 1.6 m tall, weighed about 50 kg and be approximately 46 years old.

Imaging investigations including X-rays, CT scans and endoscopy have also shown that the Icemen had experienced possible illnesses in his lifetime and had identifiable areas of musculoskeletal injury and arthritis (Murphy et al. 2003).

It has been identified that the Iceman has a laceration between the right index finger and right thumb possibly related to fighting, and he has an arrow wound in the left scapula. X-ray and CT shows that the arrow is lodged under the left scapula and there is a large haematoma present (Gostner and Egarter Vigl 2002). While this arrow injury did not kill him outright, it most certainly contributed to his death—most likely from blood loss, exposure and shock. Even in modern medical settings more than 60 % of such injured people die before reaching any hospital based treatment (Pernter et al 2007).

Two of us (WFK, MK) have performed a clinical examination of the Iceman, and have studied the available photographs and imaging pertaining to the musculoskeletal system of the Iceman. The skin of the Iceman has numerous linear non-decorative carbon tattoos. These have been presumed to possibly be “medicinal” tattoos impregnated into the skin for therapeutic reasons, and may have been used to administer acupuncture-like treatment for pain (Dorfer et al. 1998; Fleckinger 2005; Pabst et al. 2009). Spinal imaging has provided clues as to the possible sites of pain and hence sites for administration of the “medicinal” tattoos.

We contend that “medicinal” tattoos may be evidence for the presence and location of chronic or persistent pain sites in the Iceman’s musculoskeletal system.

## Methods and results

### Data analysis

A literature search has been conducted by us over the past 7 years. This has included publications from PubMed, ISI Web of knowledge, text books on the Iceman; Google search for scholarly articles; publications in archaeology journals. Only texts in English or with English translations have been used.

Key words included: Iceman, Similaun Iceman, Ötzi, transitional vertebra.

All of us have studied photographs, X-rays and CT scans and published literature pertaining to the Iceman.

### Clinical examination

Two of us (WFK and MK) first saw the Iceman in 2004, and in 2005, with the generous permission of Dr. Egarter-Vigl, Chief Pathologist for the Iceman, we performed a clinical examination of the musculoskeletal system. The Iceman is preserved at −6 °C in 98 % humidity and in this state preserved rigidly frozen. It was therefore possible to examine all digits, limbs, and the spine, but we could not perform any standard clinical joint range of motion, of the digits, limbs and spine.

We were able to view and document the location of the numerous non-decorative carbon impregnated tattoos.

### Ethical approval and advice

Approval to undertake examination of the Iceman and access to records was obtained from the Dr. A. Fleckinger, Director of the South Tyrol Museum of Archaeology, Bolzano, Italy in 2005. We also obtained clinical and anthropological advice from Dr. Eduard Egarter-Vigl, Chief Pathologist (em), Dr. Paul Gostner, Chief Radiologist (em), Bolzano, and Professor Albert Zink, Head of the EURAC-Institute for mummies and the Iceman (European Academy), Bolzano, Italy.

## Results and discussion

Our conventional musculoskeletal clinical examination did not reveal any visible or palpable findings of bone and joint disease or injury the Iceman may have acquired in life.

**Fig. 1:**
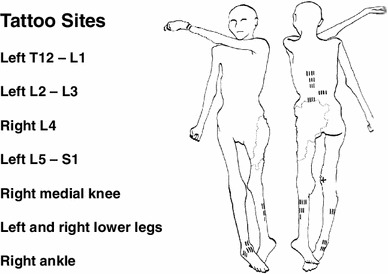
A graphic outline of the prone and supine positions of the Iceman. The small linear strokes identify the approximate locations of skin impregnated carbon tattoo sites. Adapted from Fleckinger (2005)

**Fig. 2:**
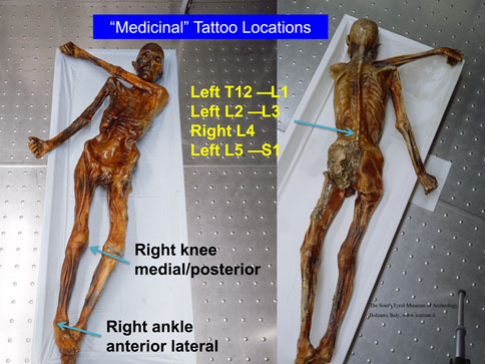
The prone and supine position of the Iceman with the *arrows* showing location of “medicinal” tattoo sites at: vertebrae thoracic 1 (T1) and lumbar (L) levels 1, 2, 3, 4, 5, and Sacral (S) 1; the *right* medial posterior knee; and *right* anterior lateral ankle

**Fig. 3:**
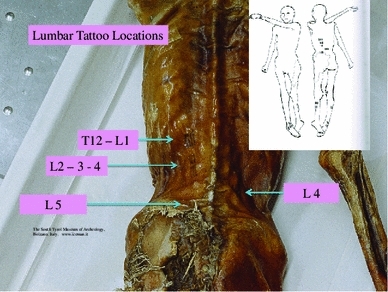
Prone position of the Iceman, which shows the tattoos in the region of the lumbar spine and adjacent thoracic and sacral areas

**Fig. 4:**
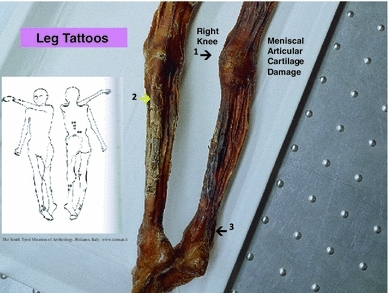
Position of leg tattoos at: *1* the *right* knee, which may represent medial meniscal cartilage and or articular cartilage damage, and/or medial collateral ligament damage, *2* the level of gastrocnemius and soleus muscles, which are common sites of expression of sciatic radicular pain in humans, and *3* the *right* ankle tattoo sites which may represent local ligamentous structure damage

**Fig. 5:**
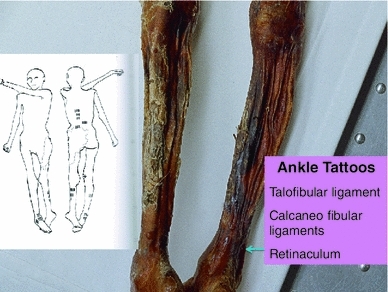
Position of ankle tattoos, which may represent local ligamentous structure damage at any or all of the talofibular ligament, calcaneo fibular ligaments, and retinaculum. The ankle region is also a common site of expression of sciatic radicular pain in humans

The tattoos on the Iceman’s back were present on the left of the mid to lower spine adjacent to thoracic vertebra 12 (T12) and lumbar vertebra 1 (L1): to the left at L2 to L3; on the right at L4; and on the left at L5 and sacral 1 (S1). There were also tattoos on the legs at the right posteromedial knee, on the right and left posterior lower legs over gastrocnemius and soleus muscle areas, and at the anterior and lateral right ankle. There is also a circular stain around the left wrist which may or nor be a tattoo (Fleckinger 2005; Pabst et al. 2009) (Figs. 1, 2, 3, 4, and 5—tattoo sites).

### Imaging

Several musculoskeletal abnormalities have been identified on X-ray and CT and have been reported by others (Gostner and Egarter Vigl 2002; Murphy et al. 2003)—(Table 1). These have included, osteoarthritis of the facet joints of the neck, osteoarthritis of the right hip, healed rib fractures, frostbite lesion of the left little toe, and an arrow wound to the left scapula (Gostner and Egarter Vigl 2002; Murphy et al. 2003; Gostner et al. 2011)—(Table 1).

**Table 1:** Musculoskeletal findings on the Iceman

Cervical facet joint damage
11 large rib bearing thoracic vertebrae
Fractures left ribs 5–9
Osteoarthritis inferomedial right hip joint
Frostbite—left little toe
Arrow injury–to left scapula

Source: Murphy et al. (2003) and Gostner and Egarter Vig (2002)

**Fig. 6:**
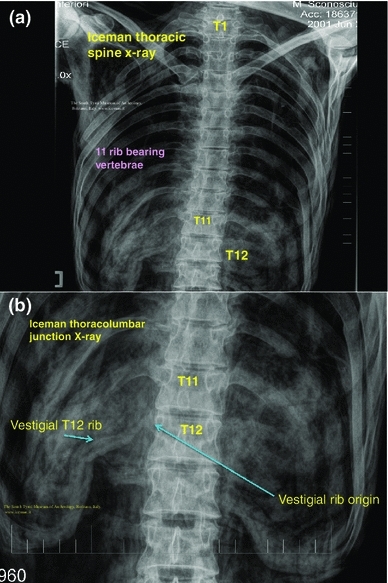
**a** Iceman thoracic spine X-ray which shows the thoracic vertebrae with 11 normal sized *right* and *left* ribs. The location of thoracic (T) vertebrae 1, 11 and 12 are shown as T1, T11, T12, **b** the X-ray of the lower thoracic spine and upper lumbar spine known as the thoracolumbar junction is shown to identify origin and position of the *right* thoracic 12 vestigial rib

**Fig. 7:**
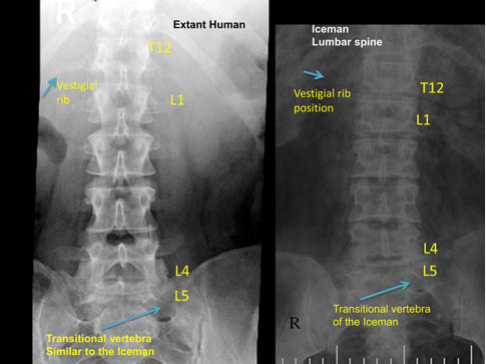
Iceman anteroposterior lumbar spine X-rays in comparison to an extant human with the same configuration of a right side thoracic 12 vestigial rib and lumbar 5 transitional vertebra. The Iceman’s vestigial rib is not as clearly seen in this image as in Fig. 6b

**Fig. 8:**
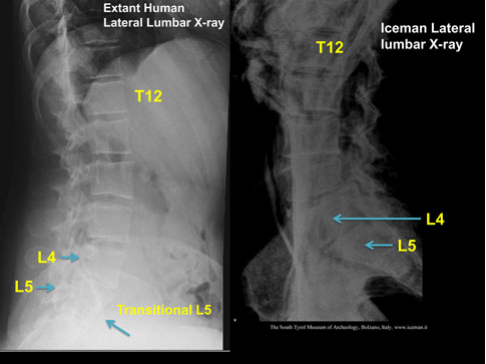
Iceman lateral lumbar X-rays in comparison to an extant human with the same configuration of a lumbar 5 transitional vertebra. The transitional L5 of the extant human and the Iceman are incorporated in the upper sacrum

**Fig. 9:**
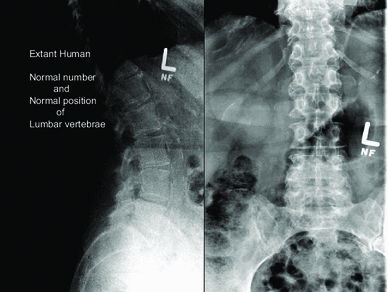
Extant human lumbar X-rays which show the normal number (5) and position of the lumber vertebrae

**Fig. 10:**
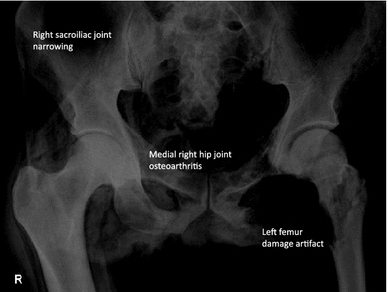
Iceman pelvis and hip joints: this shows *right* hip medial joint space loss and juxta articular sclerosis of osteoarthritis; there is narrowing of the sacroiliac joints; the *left* hip abnormalities are due to artifact when the body was extracted from the glacier ice pool

In addition, Murphy et al. (2003) described 11 rib bearing thoracic vertebrae. However, we have observed that the thoracic vertebra 12 (T12) has a small vestigial type right rib, and thus is a 12th rib—(Fig. 6a, b). This is an important anatomical finding in relation to numbering the lumbar spine, as review of the Iceman’s lumbar vertebra 5 (L5) shows that it is positioned lower than normal below the pelvic brim and incorporates with the sacrum to form the upper part of the sacrum. Thus, the Iceman’s L5 has assumed a transitional position between the lumbar spine proper and the sacrum (Murtagh and Kean 2008). This transitional L5 vertebra is seen in the anteroposterior lumbar X-ray view Fig. 7, and in the lateral lumbar X-ray view—(Fig. 8), in comparison to an extant human who has a similar lumbar 5 transitional vertebra shape and location abnormality. As a comparator in another extant human, Fig. 9 shows X-ray images of the normal number and position of lumbar vertebrae. The Iceman’s lumbar 4 to lumbar 5 disc (L4/5) and L5 to sacral 1 disc (L5/S1) appear narrow consistent with loss of disc space (Fig. 7). The Iceman’s sacroiliac joints appear narrow and more sclerosed on the right (Figs. 7, 10).

Medial joint space narrowing consistent with osteoarthritis of the right hip joint (Buchanan and Kean 2002; Adatia et al. 2012) is seen in Fig. 10.

## Discussion

The Iceman’s skin has numerous carbon, non-decorative tattoos. It has been hypothesized that these are “medicinal” tattoos that were impregnated into the skin to identify areas of pain and hence possibly assist with in the administration of “treatment”, such as a form of ancient acupuncture (Dorfer et al. 1998; Pabst et al. 2009). The “tattoo” site on the left wrist may not be a carbon tattoo but could have been a stain from leather, corresponding to the leather of the archer’s wrist guard. Murphy et al. (2003) described the cervical spine facet joint damage, right hip osteoarthritis, left rib healed fractures numbers 5–9; and left little toe frostbite. The medicinal tattoos are not located near any of these musculoskeletal sites described by Murphy et al. (2003). It is not unusual for cervical facet abnormalities to be asymptomatic. Rib fractures, although painful in the acute stage, are not painful when healed. Frostbite of the left little toe would also be painful in the acute stage but would not be painful once healed. Although patients with the same degree of right hip osteoarthritis as the Iceman may exhibit groin pain, lateral and posterior hip girdle pain, and pain in the hip region with walking, many patients with this degree of joint damage can be asymptomatic (Kean et al. 2004; Adatia et al. 2012).

If the “medicinal tattoo” hypothesis is correct, this implies that the tattoo sites on the lower thorax, lumbar areas, posteromedial right knee, lower legs, and right ankle may be sites of chronic/persistent or recurrent pain.

There are no observable major arthritis changes in the right knee X-ray, but the tattoo site may be the location of pain generated by medial collateral ligament damage and medial meniscal damage. His belongings suggest he was an archer. If he adopted the right knee kneeling pose used by hunting archers this may have been the precipitant of chronic right knee pain related to ligament and/or meniscal damage. Further studies of the right knee with CT scans would be helpful to investigate these issues. The right ankle tattoo sites may be referred pain sites from the sciatic nerve as discussed below. The ankle tattoo sites may be local injury. Although we have no histological nor other evidence for diagnostic support, we believe based on clinical experience that it is possible that the ankle tattoos mark the site of right ankle retinacular injury, calcaneo fibular ligament injury, or more likely anterior talofibular ligament injury, which had resulted from a foot/ankle inversion injury. As stated, the Iceman was a long bow archer. When the right knee kneeling position is assumed to shoot a long bow, the right ankle would be in the fully flexed and inverted position. If this position of the right knee and ankle was assumed frequently on a long-term basis (for hunting through his lifetime) it could account for chronic strain to the ankle ligaments.

The narrow and sclerosed right sacroiliac joint has raised several questions in our undergraduate and postgraduate teaching presentations over the years. We have reviewed the right sacroiliac joint X-rays with clinical rheumatologists and radiology colleagues at McMaster and Pittsburg Universities, and our consensus feeling is that the films show right sacroiliac joint secondary osteoarthritis (degenerative) changes (Resnick et al. 1977). Ankylosing spondylitis is rarely unilateral in the sacroiliac joint—it is usually bilateral, with erosions and a symmetric sclerosis that extends superiorly with syndesmophyte formation, joint space narrowing, sclerosis, and subchondral cyst formation at different stages of the disease (Resnick et al. 1977). We do not know if HLA B27 was identified in the genome, but this and other aspects relating to the genomic associations with osteoarthritis and ankylosing spondylitis are currently under investigation.

With respect to the other more common conditions of which we have experience, and which may be associated with a unilateral sacroiliitis (Smeija et al. 2001), it is our understanding that there is no current evidence from Iceman gastrointestinal endoscopy studies that he had an inflammatory bowel disease. There are no apparent skin lesions which may resemble psoriasis, notwithstanding that any psoriasis plaque could have been eroded with time or as in some patients is minimal despite the degree of joint involvement. It is possible based on his lifestyle that he may have had at some time a reactive like arthritis of the Reiter’s or other acquired microbial types to explain the localized sacroiliac joint damage. We have no evidence to support these speculations. In the radiological article by Murphy et al. (2003), in their section of pelvis and hips, these authors stated in the last line of page 14—“No evidence of inflammatory arthropathy was detected”.

Since the human skeleton drawings by Vesalius and observations in Gray’s anatomy it has been shown that the most common numbers of human vertebrae are 7 cervical, 12, thoracic, 5 lumbar, 5 sacral and 4 coccygeal (Vesalius and Andreas 1998; Gray’s Anatomy 1949). The original Iceman’s X-ray imaging observation of only 11 rib bearing thoracic vertebrae by Murphy et al. (2003), was therefore a valuable clue to our further findings of the vestigial type right rib at T12. Once we identified T12, it required a simple count on X-ray of the 5 lumbar vertebra to identify that L5 was in a transitional position, in that it sat low in relation to the pelvic brim, was positioned on the upper sacrum, and thus formed an integral part of the upper sacrum. The transitional vertebra was initially briefly referred to by Murphy et al. (2003). We also observed that the Iceman’s L5 vertebra also had an abnormal shape. We then observed that the L4/5 and L5/S1 disc spaces were narrow; and that the right sacroiliac joint (discussed above) was narrowed and sclerosed. These spinal and pelvic abnormalities are important clues to the clinicopathophysiological musculoskeletal conditions which may have been experienced by the Iceman (Kean et al. 2004; Murtagh and Kean 2008). The condition of transitional type lumbar vertebrae with its X-ray, CT and MRI imaging findings and associated clinical features has been described by many authors over the past 90 years (Bertolotti 1917; Castellvi et al. 1984; Murtagh and Kean 2008; Konin and Walz 2010).

Back pain of various types and transitional lumbar vertebra has sometimes been referred to as Bertolotti syndrome (Bertolotti 1917). As reviewed by our clinical research unit (Murtagh and Kean 2008) and by others (Konin and Walz 2010) there have been many literature discussions, which have disputed a direct association with transitional vertebrae and back pain (Peterson et al. 2005). However, we believe that the body of clinical evidence does support an association between transitional vertebrae and episodes of spinal pain and referred pain to the legs (Murtagh and Kean 2008).

There have been several variations of abnormal shape and position described for L5, which vary from: a simple abnormality such as increased size of one or both the lumbar transverse processes; fusion or direct incorporation of the transverse process to the sacrum; incorporation of the vertebral body of L5 on to the sacrum with a vestigial lumbar 5 to sacral 1 (L5/S1) disc; and sometimes abnormal articulation of the lateral aspect of L5 with the sacrum and/or ilium to form a pseudoarticulation (Castellvi et al. 1984). The studies by Castellvi et al. (1984), and Konin and Walz (2010), have provided excellent examples and discussion of the abnormal L5 shape, position and techniques for imaging. Transitional vertebrae of the lumbar and sacral areas are easily diagnosed with standard anteroposterior and lateral X-ray views of the lumbar spine, which also include the lower thoracic vertebrae and the upper sacrum (Murtagh and Kean 2008). A thoracic spine X-ray is also of value to verify the 12 rib bearing thoracic vertebrae. When there is a transitional lumbar vertebra, T12 can have normal sized ribs but more often has small or vestigial ribs. In some cases, the vestigial ribs are only visible with a magnifier. It is our opinion, as in the case of the Iceman, that CT scan is not necessary for the diagnosis of transitional vertebra. In our investigation of patients with this condition, CT scan or MRI scan does not add to the majority of clinical management interventions for the back pain, and for the management of any radicular or referred pain to the legs (Murtagh and Kean 2008). CT scan and or MRI scan, in addition to fluoroscopy, may be of value to interventional pain specialists for accurate administration of a nerve block type injection. Similarly CT scan and or MRI scan may be of value for certain proposed therapeutic surgical procedures.

**Fig. 11:**
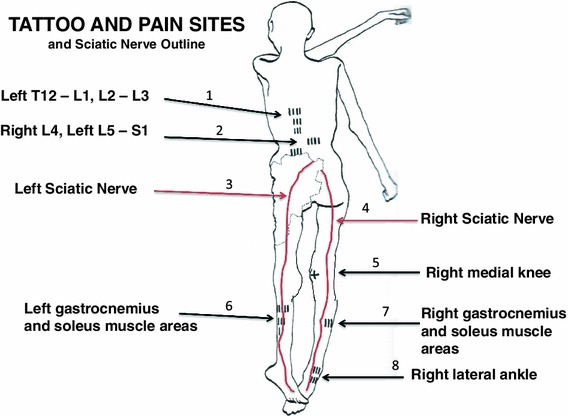
A graphic outline of the prone and supine positions of the Iceman is shown. The small linear strokes identify the approximate locations of skin impregnated carbon tattoo sites adapted from Fleckinger (2005). This image has been overlayed with a linear caricature of the *left* and *right* sciatic nerves from their origin in the lumbar 3, 4, 5 and sacral 3, 4, 5 nerve root positions, to the distal distribution in the posterior upper and lower legs and the ankles and feet. The gastrocnemius and soleus muscle areas and sometimes the ankles are sites of sciatic nerve radicular pain expression in humans (Kimura 1989), and may provide some explanation of the location of these presumed “medicinal” tattoos

We have identified from our own research studies (Murtagh and Kean 2008) and clinical observations, and the literature citations above that a transitional L5 vertebra can result in mechanical lumbar back pain and related sciatic nerve referred pain. The sciatic nerve is derived from the lumbar L4, 5, sacral 1, 2, 3 nerve roots and radiates through the buttock area to the leg and foot (Kimura 1989). It is most likely that the tattoos in the left T12 to L1 and L2 to L3 thoracolumbar area correspond to sites of chronic mechanical back pain. The right L4 and left L5/S1 adjacent lower lumbar tattoos correspond to the problem of chronic lower lumbar mechanical back pain possibly related to the disc and facet joint narrowing at L4/5 and L5/S1 (Figs. 1, 2, 3, 4, 5). The bilateral lower leg tattoos in the calf gastrocnemius and soleus muscle area correspond to possible sites of sciatic nerve referred pain in an individual with mechanical back pain who had episodes of sciatica. In addition, the right ankle tattoos can also correspond to sites of referred sciatic nerve pain (Kimura 1989). The presence of the lumbar and leg tattoos are consistent with therapeutic sites for the treatment of chronic thoracolumbar pain and lumbar mechanical back pain with sciatic nerve referred pain and radicular pain related to the disc and facet joint narrowing and transitional vertebra of the Iceman (Fig. 11).

We conclude that the presence of the back and leg medicinal tattoos indicate that when the Iceman was alive, he would have had episodes of chronic persistent mechanical back pain, and also episodes of pain to the lower legs and possibly the right ankle due to referred pain and/or sciatic radicular pain of varying severity (Kean et al. 2004; Kimura 1989; Murtagh and Kean 2008).

Chronic musculoskeletal non-cancer pain is usually described in terms of the time, such as greater than 6 months for continual or intermittent perceived painful episodes. Chronic musculoskeletal pain is a complex series of symptoms, signs and often psychosocial behavioural processes (Kean et al. 2004, 2008; Michel et al. 2009). In many cases, the process of chronic pain is the continuation of the pain response beyond that, which is physiologically necessary to warn the host. Although this is also an imprecise concept since the host needs to be aware of all actions which might result in further tissue damage or dysfunction.

There is often poor correlation with joint/disc damage symptoms, and related imaging appearance (Kean et al. 2004). The presumed source of joint/disc pain stems from the synovium, subchondral bone, capsule, periosteum, and adjacent nerves. Joint/disc damage generates nociceptive stimuli (Pelletier et al. 2001). Many patients with joint or spinal disc damage, experience episodes of pain in the area of the joint and disc damage but can also suffer pain referred in the distribution of the related nerves. The pain is not necessarily persistent and can vary in character, distribution, duration and intensity. Since in many circumstances, there can be a poor correlation between joint/disc damage and clinical findings, the imaging needs to be correlated with clinical signs and symptoms, (Kean et al. 2004).

Osteoarthritis (OA) and mechanical back pain of the kind that the Iceman may have experienced are the most common musculoskeletal disorders worldwide, characterized by cartilage/disc matrix destruction by proinflammatory mediators (Pelletier et al. 2001; Dreier 2010). The biochemical mechanisms and functional pathways of chronic musculoskeletal pain involve both peripheral and central components of pain stimulation related to damage or deformed bones and joints, tendons, ligaments, cartilage and related nerve supply. From the point of view of potential aetiology with modern insight we can speculate that the Iceman would have experienced peripheral and central nervous system components of acute and chronic pain resulting from cyclooxygenase (COX)-2 and COX-1 derived prostaglandin E_2_ (PGE_2_), nitric oxide (NO), nerve growth factor (NGF), substance P and calcitonin gene-related peptide (CGRP), and ATP (Rodger 2009). However, in chronic musculoskeletal pain there are no clinical signs to serve as indicators, but the functional joint changes may have involved production of cytokines such as interleukin-1 (IL-1) and interleukin-6 (IL-6), which amplify the production of PGE_2_ and NO by induction of COX-2, and inducible nitric oxide (iNOS) enzymes, respectively. Also cytokines may have mediated joint injury. Chronic musculoskeletal pain often involves neuropathic and neuro-inflammatory changes underlying dull throbbing pain that principally involves activation of non-myelinated C-fibre pathways (Stucky et al. 2001; De Leo et al. 2006; Hansson 2006; Pruimboom and van Dam 2007).

If we had been able to interview the Iceman in his later lifetime, he would most likely have expressed the symptoms of chronic mechanical pain in the thoracolumbar and lumbar areas, and episodes of referred pain to the lower legs in a sciatic nerve distribution. He would also have expressed that he had episodes of medial to posterior right knee pain with walking. He would have had episodes of right ankle pain, worse with walking. In addition to the low back pain and leg pain, he may have described back ache with spinal stiffness of variable intensity and duration; para lumbar and leg muscle spasm with associated pain, which may be dull or even “electric” or nerve like in nature; numbness and tingling in the back, buttocks and legs; and leg and ankle weakness.

If we were able to observe the Iceman, the clinical signs could have included: an abnormal slow moving gait with possible limp related to the low back, the right hip, right knee or right ankle or a combination of any or all of these. We may have been able to detect low back tenderness to clinical palpation. We may observed that he had pain in a sciatic nerve distribution when he was asked to perform a right or left straight leg raise while lying on his back: a standard test of sciatic pain. He may have had local right lateral ankle tenderness and pain on movement at the retinaculum, calcaneofibular ligament, and anterior talofibular ligament. If there was significant nerve root compression/irritation at L4/5 and L5/S1 he may have had possible loss of ankle dorsiflexion power with foot drop (lumbar 5 nerve dysfunction) and/or absence or diminished ankle reflex (sacral 1 nerve dysfunction). We may have also observed possible lower leg calf gastrocnemius and soleus muscle atrophy.

As to the aetiology of the Iceman’s joint condition it is only possible to speculate based on recent genome sequencing of his DNA (Keller et al. 2012). These observations show that he has evidence of predisposition to coronary heart disease. Atherosclerosis and other vascular conditions are common in patients with osteoarthritis (Jonsson et al. 2009; Hoeven et al. 2012) thus perhaps the predisposition to coronary heart disease was contributory to some of the osteoarthritic joint damage.

The genome sequencing of his DNA (Keller et al. 2012) also identified that he had been infected with the spirochete, *Borrelia burgdorferi,* the causative pathogen of Lyme disease (Lantos 2011; Rashid and Ebringer 2012). The *Borrelia burgdorferi* spirochete is transmitted by the ixodid tick, of the Ixodes ricinus complex, and the Lyme disease is thus most prevalent in rural areas, which are the habitat of deer and deer ticks, and the habitat of Ötzi. Lyme disease is a multisystem illness which is expressed in both children and adults (Steere et al. 1987). A large red rash occurs in about 80 % of patients after an incubation of 3–32 days. Secondary smaller skin lesions occur within a few days and are accompanied by headache, neck stiffness, chills, fever, malaise, myalgias and arthralgias. In untreated individuals, approximately 10 % have cardiac involvement and 15 % have neurological involvement including encephalopathy, and sensorimotor radiculopathies. Musculoskeletal symptoms are usually tendon and bursal pain, and migratory joint pains and swelling, especially the knees. About 60 % of untreated patients develop chronic arthritis, and about 10 % develop cartilage damage and bone erosions (Steere et al. 1987). The joint arthritic symptoms are associated with the production of IL-17 and IL-23 (Oosting et al. 2011), both of which are amongst the key cytokines involved in the immuno-pathogenesis of rheumatoid and other auto-immune arthropathies (Rashid and Ebringer 2012). Whether Ötzi was symptomatic at any time in his life with some or several manifestation of Lyme disease, cannot be known, but evidence that he was infected with Lyme disease is an additional possible contributory factor to possible joint pain and joint damage.

There have been some suggestions that Ötzi may have taken medicinal plants e.g., blackthorn for vitamins, hop hornbeam and other natural products (Dickson 2011). Some preparations may have been taken for intestinal parasitic infections (Dickson 2011), which of themselves may have had systemic inflammatory activity. Despite the evidence described above, which suggests he had mechanical spinal and joint pain, neuro-radicular type pain, and possibly could have had episodes inflammatory joint pain, we have no knowledge that he took oral agents as a treatment for joint pain.

However, we do believe that the permanence of the medicinal tattoos was therapeutic target sites for chronic pain management.

## Conclusion

The Iceman had many well documented sites of musculoskeletal damage acquired in his lifetime which included: osteoarthritis of the facet joints of the neck; osteoarthritis of the right hip; healed left 5–9 rib fractures; frostbite lesion of the left little toe; and an arrow wound to the left scapula. The Iceman had numerous non-decorative carbon tattoos. It is hypothesized that these “medicinal” tattoos identified sites of possible chronic or persistent pain, and hence possibly assisted in the administration of “treatment”, such as a form of ancient acupuncture (Dorfer et al. 1998; Pabst et al. 2009). We identified that lumbar vertebra L5 was transitional in that it sat lower than normal in relation to the pelvic brim, was positioned on the upper sacrum, and thus formed an integral part of the upper sacrum (Murtagh and Kean 2008). The left T12 to L1 and L2 to L3 tattoos likely correspond to areas of chronic mechanical back pain. The right knee posteromedial tattoo likely corresponds to chronic medial meniscal and medial collateral ligament pain. The right ankle tattoos likely correspond to chronic pain at any or all of the retinaculum, calcaneofibular ligament, anterior talofibular ligament, but most likely the last. We contend that the right L4 and left L5/S1 lower lumbar tattoos correspond to sites of lower lumbar mechanical back pain possibly related to the disc narrowing at L4/5 and L5/S1, and local nerve root impingement and irritation, and the bilateral lower leg tattoos over gastrocnemius and soleus in the calf muscle area (and possibly the right ankle) correspond to sites of chronic sciatic nerve referred pain. We presume that he would have had chronic musculoskeletal pain of a dull throbbing type that principally involves activation of non-myelinated C-fibre pathways with activation of neuro-immune systems such as the microglial cells.

We contend that the Iceman may have exhibited the physical signs of right knee pain: right ankle pain; and most principally chronic mechanical back pain and sciatic nerve root irritation which include leg referred pain, leg and ankle weakness, leg tingling and numbness. During the episodes of chronic low back and leg pain he may have walked with a limp.

The presence of atherosclerosis and its association with osteoarthritis: infection with Lyme disease; and X-ray evidence of osteoarthritic joint abnormalities; and spinal damage, are convincing evidence that Ötzi had joint and spinal pain at some times in his life, and especially in his latter years. We conclude that the locations of the tattoos at the right posterolateral knee, right anterolateral ankle, left thoraco lumbar spine, lower lumbar spine and both calf gastrocnemius and soleus areas were therapeutic treatment sites for the management of chronic pain.
